# Analysis of the diagnostic efficacy of targeted next-generation sequencing for tuberculosis in patients with HIV

**DOI:** 10.3389/fcimb.2026.1803293

**Published:** 2026-04-29

**Authors:** Chang Song, Ying-Xing Nong, Chun-Yan Zhao, Xue-Wen Huang, Ai-Chun Huang, Chun-Ming Gong, Wei-Wen Li, Qiu-Qing Tan, Zhen-Tao Huang, Xiao-Shi Lin, Chun-Mei Zeng, Qing-Dong Zhu

**Affiliations:** 1 Department of Tuberculosis, The Fourth People’s Hospital of Nanning, Nanning, Guangxi, China; 2 Clinical Medical School, Guangxi Medical University, Nanning, Guangxi, China; 3 Department of Medical, The Fourth People’s Hospital of Nanning, Nanning, Guangxi, China

**Keywords:** diagnostic efficacy, HIV, targeted next-generation sequencing, tuberculosis, Xpert

## Abstract

**Background:**

To evaluate the diagnostic performance of targeted next-generation sequencing (tNGS) for tuberculosis (TB) in patients with human immunodeficiency virus (HIV) and to compare it with traditional diagnostic methods.

**Methods:**

A total of 66 HIV patients were enrolled, including 25 with confirmed TB infection and 41 without TB. Statistical analyses were performed using SPSS 22.0. Diagnostic performance was assessed using the chi-square test. Sensitivity, specificity, positive predictive value (PPV), negative predictive value (NPV), Kappa coefficient, and the area under the curve (AUC) of the receiver operating characteristic curve (ROC) were calculated for tNGS. McNemar’s chi-square test was used to compare the diagnostic efficacy of tNGS with other methods.

**Results:**

The sensitivity, specificity, PPV, NPV, AUC, and Kappa of tNGS were 76.00%, 100.00%, 100.00%, 87.20%, 0.880, and 0.797 respectively, all superior to traditional methods. When combined with Xpert, the sensitivity increased to 84.00%, and the AUC improved to 0.920, indicating strong diagnostic complementarity.

**Conclusion:**

tNGS exhibits high sensitivity and specificity for diagnosing TB in patients with HIV. Notably, the combination of tNGS with Xpert further enhances diagnostic efficacy, underscoring its potential clinical promotion value.

## 1 Introduction

Tuberculosis (TB), a chronic infectious disease caused by Mycobacterium tuberculosis (MTB), remains one of the leading global public health challenges (Trajman et al., 2025). The “2025 Global TB Report” reveals that TB remains one of the world’s deadliest infectious diseases. Data show that in 2024, an estimated 10.7 million people fell ill with TB, and over 1.2 million died from the disease. The burden of the disease is highly concentrated, with eight countries—India (25%), Indonesia (10%), the Philippines (6.8%), China (6.5%), and Pakistan (6.3%)—accounting for 67% of the global total. Additionally, a meta-analysis based on a random-effects model indicates that the pooled prevalence of TB/HIV co-infection is 14%, with significant regional variation. The burden is highest in Africa, where the prevalence reaches 22%, compared to 7% in Asia (Wu et al., 2025). Co-infection with HIV significantly exacerbates the global TB burden (Shen, 2024). The immunosuppressed state of HIV-infected individuals increases their risk of TB infection, accelerates disease progression, and enhances the likelihood of transmission (Rego de Figueiredo et al., 2021). Among these individuals, the progression from latent TB infection to active disease occurs more rapidly (González Fernández et al., 2020). In 2023, there were approximately 662, 000 new cases (6.1%) of patients co-infected with HIV, and of the 1.25 million TB deaths, 161, 000 occurred in patients with HIV (World Health Organization, 2024). However, autopsy studies suggest that up to 45.8% of patients who died from TB had not received a clear definitive diagnosis at the time of death (Gupta et al., 2015). Early and accurate diagnosis is therefore essential to transmission and mortality. However, in HIV-TB co-infected patients, routine TB methods including smear microscopy, culture, exhibit low sensitivity due to the patients’ low immune function and low bacterial loads (Yang et al., 2022). In addition to the traditional diagnostic methods mentioned above, the tuberculin skin test (TST) and interferon-gamma release assay (IGRA) remain important immunological tools for screening latent tuberculosis infection and assisting in the diagnosis of active tuberculosis. It is worth noting that a Cochrane systematic review (CD016070), conducted by the Department of Infectious Diseases and Tropical Medicine at the University Hospital of Munich in collaboration with international partners, has confirmed that the simultaneous use of low-complexity nucleic acid amplification testing on respiratory specimens and stool samples significantly improves the pathogen detection yield in childhood tuberculosis compared to single-method testing. Furthermore, the study indicates that adding urine lipoarabinomannan detection (LF-LAM) can further enhance diagnostic accuracy in children living with HIV. However, due to immunosuppression, the clinical manifestations of TB in HIV-infected individuals are usually atypical, such as fever, weight loss, and lymphadenopathy, closely resembling other opportunistic infections and complicating accurate diagnosis. It has been well established that HIV-1 infection alters the course of Mycobacterium tuberculosis infection and significantly increases the risk of developing active tuberculosis. Conversely, tuberculosis also promotes HIV-1 replication, transmission, and genetic diversity, creating a mutually reinforcing pathological relationship between the two pathogens during co-infection that synergistically drives disease progression (Bell and Noursadeghi, 2018). Meta-analyses have demonstrated that the risk of multidrug-resistant tuberculosis (MDR-TB) is increasing among HIV-positive individuals, with a particularly significant rise in risks associated with primary drug resistance transmission (Sultana et al., 2021). In this context, rapid diagnostic methods have become an urgent necessity for guiding early clinical medication, while the low bacterial load resulting from HIV-induced immunodeficiency significantly limits the sensitivity of traditional diagnostic approaches. In addition, the proportion of extrapulmonary TB cases is significantly higher in HIV-infected individuals compared to the general population. Common forms include lymph node TB, tuberculous meningitis, and skeletal TB, all of which are more challenging to diagnose (Saita et al., 2021). For example, the incidence of tuberculous meningitis is notably elevated in HIV-infected individuals, and it carries a high mortality rate, especially when diagnosis and treatment are delayed (Kimuda et al., 2023). These challenges highlight the urgent need for rapid and accurate diagnostic method for TB in patients with HIV.

In recent years, the development of next-generation sequencing (NGS) technology has opened new possibilities for TB diagnosis. NGS offers high throughput, sensitivity, and resolution, enabling simultaneous detection of multiple pathogens and their drug-resistant gene mutations, providing comprehensive data for clinical decision-making (Chen et al., 2024). Among NGS approaches, targeted next-generation sequencing (tNGS) enhances detection accuracy by focusing on specific gene regions, while also reducing cost and simplifying data analysis (Chen et al., 2024). Emerging studies have demonstrated the potential of tNGS in the diagnosis of TB (Murphy et al., 2023; Tao et al., 2024; Zhang et al., 2024; Chen et al., 2025; Yang et al., 2025a; Yang et al., 2025b). It is worth noting that current research has largely focused on immunocompetent populations. In HIV-positive individuals, tNGS has been more frequently applied to detect HIV drug resistance (Bonifacio et al., 2022; Ouyang et al., 2024), while studies specifically addressing HIV and Mycobacterium tuberculosis co-infection remain relatively scarce. HIV-infected patients are characterized by immunocompromised status and low pathogen loads, which limit the sensitivity of traditional diagnostic methods. This study addresses this gap by systematically evaluating the diagnostic performance of tNGS in HIV patients with tuberculosis co-infection, thereby providing new evidence-based insights to optimize diagnostic strategies for this population.

Therefore, this study aims to systematically evaluate the diagnostic performance of tNGS for detecting TB in patients with HIV. and exploring its application value in different clinical scenarios have important scientific and practical significance. Key metrics, including sensitivity, specificity, positive predictive value (PPV), and negative predictive value (NPV), will be assessed and compared to traditional diagnostic methods. The goal is to identify a more accurate and efficient diagnostic strategy to improve TB detection in this vulnerable population.

## 2 Materials and methods

### 2.1 Inclusion of test samples

This retrospective study included patients suspected of HIV co-infected with TB who presented to the Fourth People’s Hospital of Nanning between October 2021 to June 2025 (**Table 1**). Diagnostic tests performed included Acid-fast staining (AFB), TB-DNA detection, mycobacterial solid culture, and tNGS. This study complied with the ethical standards of the Declaration of Helsinki and was approved by the hospital’s ethics committee (Approval Number: [2023]24). All identifiable patient information was anonymized. Informed consent was waived due to the retrospective design. Inclusion criteria were as follows: (1) Confirmed HIV infection through enzyme-linked immunosorbent assay and Western blot or immunofluorescence, or clinical AIDS diagnosis by a qualified institution. All HIV-positive patients included in this study were receiving antiretroviral therapy. (2) Presence of clinical features suggestive of TB, such as persistent cough > 2 weeks, fever > 38 °C, night sweats, weight loss > 5% within three months, or radiological evidence (e.g., pulmonary nodules, infiltrates, or cavities). (3) Complete clinical records and compliance with diagnostic procedures through to confirmation or exclusion of TB. The exclusion criteria included: (1) Confirmed alternative lung diseases (e.g., lung cancer, bacterial pneumonia, lung abscess, or bronchiectasis) without evidence of TB. (2) Anti-TB treatment within the previous three months. (3) Complicated such as severe hepatic or renal insufficiency, acute cardiovascular or cerebrovascular disease, or active hematological malignancies. (4) Psychiatric conditions (e.g., schizophrenia, severe depression) or cognitive impairments that impeded cooperation. (5) Pregnancy or lactation.

**Table 1:** Comparison of clinical characteristics between TB and non-TB patients.

Clinical characteristics	Total(n=66)	TB(n=25)	Non-TB(n=41)
Gender			
Male	54	20	34
Female	12	5	7
Age	53.64 ± 11.95	52.84 ± 10.31	54.12 ± 12.94
Comorbidities			
NTM	2	1	1
Diabetes	3	1	2
CRP	38.36 ± 46.31	20.03 ± 4.01	55.65 ± 8.69
ESR	70.53 ± 63.43	44.00 ± 8.80	71.98 ± 11.24
CD3+	756.36 ± 559.41	479.11 ± 95.82	608.88 ± 95.09
CD4+	215.80 ± 242.11	292.82 ± 58.56	209.26 ± 32.68
CD8+	505.00 ± 402.28	302.75 ± 60.55	455.86 ± 71.19
CD4/8+	0.50 ± 0.60	0.42 ± 0.08	0.68 ± 0.11

### 2.2 Diagnostic criteria

HIV diagnosis followed the “Guidelines for the Diagnosis and Treatment of AIDS in China (2021 Edition)” (Acquired Immunodeficiency Syndrome Professional Group SoID et al., 2024). The diagnosis was based on the national standard “Diagnostic Criteria for Pulmonary Tuberculosis” (WS 288-2017). A definitive TB diagnosis required one or more of the following: Positive bacteriological or molecular evidence for MTB. Pathological confirmation, such as caseous necrosis or epithelioid granuloma formation. Clinical and radiological improvement following empirical anti-TB therapy over three months in cases with high suspicion but negative bacteriological results.

### 2.3 Sample collection for detection

Sample were collected aseptically using physical sterilization methods (e.g., steam pressure). Prior to anti-infective treatment, various clinical specimens were obtained sputum, bronchoalveolar lavage fluid (BALF), cerebrospinal fluid (CSF), effusion, and tissue samples, and subjected to AFB, mycobacterial solid culture, Xpert, and tNGS. Sputum: Patients rinsed their mouths thrice with water (≥1 min each), took a deep breath, and coughed deeply to produce 3–5 mL of sputum, which was stored at 2–8 °C. BALF: Following nasal anesthesia and lubrication, a bronchoscope was inserted. Warm saline (60–100 mL total) was instilled in five portions (20–50 mL each) and recovered under negative pressure (< -100 mmHg) into sterile bottles. Blood: 5 mL of fasting venous blood was drawn the morning after admission. Tissue samples: Obtained via percutaneous biopsy, fixed in 10% neutral buffered formalin, dehydrated, embedded in paraffin, sectioned at 4–6 µm. Effusions: Collected via ultrasound-guided puncture. CSF: Obtained via lumbar puncture using aseptic technique (2–3 mL collected). This study strictly adhered to a standardized sample collection process to ensure the applicability of diagnostic methods and the reliability of results. Sample selection was primarily guided by the patient’s clinical presentation and sample accessibility: For patients with suspected pulmonary tuberculosis who were capable of spontaneous sputum production, sputum specimens were prioritized. For those with insufficient sputum production or who were unable to expectorate, bronchoalveolar lavage fluid was collected via bronchoscopy. In cases where respiratory samples could not be obtained, or when specific forms of tuberculosis were highly suspected based on clinical findings, specimens were collected according to the site of involvement—for example, cerebrospinal fluid was collected from patients with suspected central nervous system tuberculosis. This stratified sampling strategy was designed to encompass a broad spectrum of clinical presentations while considering the suitability of each sample type for the various testing methods employed.

### 2.4 Acid-fast bacilli staining

Acid-fast bacilli were detected using fluorescent auramine O staining. Samples were processed following standard smear preparation procedure, and slides were fixed by flame. A commercial staining kit (Zhuhai Bioso Biotechnology Co., Ltd., Specification: 4×250 ml) containing three reagents was used. This kit contained three solutions, including auramine O solution (main components were auramine O and phenol), acid alcohol solution (a mixture of ethanol and hydrochloric acid), and 5% potassium permanganate solution. The staining agent was evenly applied to the smear and allowed stand for 30 minutes before rinsing gently with running water. Decolorization was performed by covering the slide with acid-alcohol for 3 minutes or until complete fading, followed by another rinse. Counter-staining was carried out by applying the potassium permanganate solution for 1 minute, followed by rinsing and air-drying. Slides were examined under a fluorescence microscope using dark-field conditions. Acid-fast bacilli appeared as slightly curved, rod-shaped organisms emitting characteristic yellow fluorescence.

### 2.5 TB-DNA detection

In this study, a mycobacterial nucleic acid detection kit (Chengdu Bio-Future Biotechnology Co., Ltd., National Medical Device Registration No. 20173401341) was used, based on duplex polymerase chain reaction (PCR) combined with TaqMan probe technology for the detection of Mycobacterium tuberculosis DNA (TB-DNA). Specific primers and probes were designed targeting the unique gene sequences of Mycobacterium tuberculosis complex (MTB) and non-tuberculous mycobacteria (NTM), with different fluorescent labels to distinguish between the two groups. By real-time monitoring of multi-channel fluorescence signals, MTB and NTM could be accurately identified simultaneously within a single reaction system. Three high-performance real-time PCR instruments were employed in this study, including the 7500 model manufactured by Applied Biosystems (USA), and the SLAN-96S and SLAN-96P systems developed by Shanghai Hongshi Medical Technology Co., Ltd. All detection reagents were provided by the aforementioned kit, which is an optimized proprietary PCR-fluorescence probe kit featuring high accuracy and specificity, suitable for molecular identification of mycobacteria.

### 2.6 Xpert MTB/RIF

According to the standard operating procedure, the Xpert MTB/RIF assay was performed using the MTB rpoB Gene Mutation Detection Kit (Real−time Fluorescence PCR method; product number: 301−3300−ZH−CN). The assay was carried out on a fully automated medical PCR analysis system (models: GeneXpert IV, GeneXpert XVI, GX−I R2, GX−II R2, GX−IV R2, GX−XVI R2, Infinity−48s, and Infinity−80). Before operation, the sterile dedicated pipette provided in the kit was used to aspirate the liquefied specimen, and the liquid level was adjusted so that the meniscus was exactly above the lowest graduation mark of the pipette. Subsequently, the lid of the test cartridge was opened, and the aspirated specimen was slowly injected into the cartridge through the sample loading port to avoid aerosol formation caused by splashing or bubble rupture. After loading, the lid of the cartridge was immediately closed and securely fastened to prevent leakage or cross−contamination during the detection process.

### 2.7 Mycobacterial culture

A 4% (w/v) sodium hydroxide (NaOH) solution was prepared by dissolving 4 g of NaOH in 100 mL of distilled water, followed by sterilized using high-pressure steam. For decontamination, 5 mL of each specimen was mixed with an equal volume of the NaOH solution, vortexed for 20 seconds, and incubated at room temperature for 15 minutes. The reaction was neutralized with phosphate-buffered saline (PBS, pH of 7.2), and the suspension was centrifuged to collect the pellet. All decontamination procedures were completed within 20 minutes of NaOH addition. The processed sample (0.1 mL) was inoculated onto modified Lowenstein-Jensen medium (Zhuhai Bioso Biotechnology Co., Ltd., Specification: 50 tubes/box) under aseptic conditions. Cultures were examined on days 3 and 7 after inoculation, and subsequently at weekly intervals for up to 8 weeks. Growth confirmed by smear microscopy within 7 days was classified as rapidly growing mycobacteria. Growth confirmed after 7 days was classified as slowly growing mycobacteria. Absence of growth at 8 weeks was reported as negative.

### 2.8 Targeted next-generation sequencing

The tNGS analysis for this study was conducted by Hangzhou Shengting Medical Technology Co., Ltd. The samples were liquefied and lysed using NALC-NaOH at a final concentration of 2%. The samples were treated by adding proteinase K, lysozyme, and plasmids. Test samples were initially added to the reagent solution, incubated, and then mixed with absolute ethanol and centrifuged (Qilinbeler/Luxiangyi, China). The supernatant was transferred back to an EP (Eppendorf) tube plate. Magnetic beads were added, followed by vortexing and standing incubation, after which the supernatant was discarded. Subsequently, 1X WB buffer was added along with ethanol, and the supernatant was again discarded. Once the EP tubes were processed, the magnetic beads were activated, followed by a static incubation. The Qubit 4.0 fluorometer was employed for nucleic acid quality assessment to confirm compliance with standards. A PCR reaction system was prepared, DNA was added, and the mixture was verified. The reaction tubes were then placed in a PCR thermal cycler (ABI/Bio-Rad, USA) to run the amplification. Upon completion, purification was performed, and the DNA libraries were subsequently extracted. The multiplex PCR products were labeled with barcodes, and the post-PCR products were stored. The PCR products underwent purification and quality control. Sequencing was then performed using the Illumina MiSeq DX high-throughput sequencing system. Data quality was filtered and aligned to identify pathogenic microorganisms and analyze antibiotic resistance genes (a sample was determined to be positive when at least one segment was successfully mapped to a known species or genus). Known resistance-associated mutation genes were derived from currently published literature: isoniazid resistance-related genes included inhA, katG, ahpC, fabG1, and kasA; rifampicin resistance-related genes included rpoB and rpoC; ethambutol resistance-related genes included embA, embB, and embC; and streptomycin resistance-related genes included rpsL, gid, and rrs. The total targeted region is approximately 12.5 kb in length, containing 112 amplicons, with an average coverage depth of more than 1000×.

### 2.9 Statistics and analysis

Data were analyzed using SPSS 22.0. The diagnostic performance of AFB, TB-DNA detection, Xpert MTB/RIF, mycobacterial solid culture, and tNGS was compared. Categorical variables were expressed as frequencies (%) and compared using the chi-square test. Two-sided P < 0.05 was considered statistically significant. The final clinical diagnosis served as the reference standard for evaluating diagnostic performance. Sensitivity, specificity, PPV, NPV, Kappa coefficient, AUC, and ROC were calculated. McNemar chi-square test was used to compare the diagnostic performance of tNGS with other methods.

## 3 Results

### 3.1 Basic information of included patients

A total of 66 patients were included in this study, comprising 25 in the TB group and 41 in the non-tuberculosis group. The cohort consisted of 54 males and 12 females, with a mean age of 53.64 ± 11.95 years. 2 patients were co-infected with NTM, and 3 had diabetes. The overall mean C-reactive protein (CRP) level was 38.36 ± 46.31 mg/L, and the erythrocyte sedimentation rate (ESR) was 70.53 ± 63.43 mm/h. In subgroup analysis, CRP was 20.03 ± 4.01 mg/L in the TB group, and 55.65 ± 8.69 mg/L in the non-TB group. ESR was 44.00 ± 8.80 mm/h in the TB group, and 71.98 ± 11.24 mm/h in the non-TB group. Immune cell analysis showed mean counts of CD3^+^ cells at 756.36 ± 559.41/μL, CD4^+^ cells at 215.80 ± 242.11/μL, CD8^+^ cells at 505.00 ± 402.28/μL, and a CD4^+^/CD8^+^ ratio of 0.50 ± 0.60. In the TB group, the values were 479.11 ± 95.82/μL (CD3^+^), 292.82 ± 58.56/μL (CD4^+^), 302.75 ± 60.55/μL (CD8^+^), and 0.42 ± 0.08 (CD4^+^/CD8^+^ ratio). In the non-TB group, they were 608.88 ± 95.09/μL, 209.26 ± 32.68/μL, 455.86 ± 71.19/μL and 0.68 ± 0.11 respectively. Specimen types collected from patients are detailed in **Table 2**, including bronchoalveolar lavage fluid (BALF, n = 46), cerebrospinal fluid (n = 13), sputum (n = 3), biopsy tissue (n = 2), and pleural effusion (n = 2).

**Table 2:** Distribution of sample types across the study cohort.

Sample type	Number of cases	Proportion
Bronchoalveolar lavage fluid	46	69.70%
Cerebrospinal fluid	13	19.70%
Sputum	3	4.55%
Biopsy tissue	2	3.03%
Pleural effusion	2	3.03%

### 3.2 Comparison of diagnostic efficacy between tNGS and traditional methods

The diagnostic performance of five methods for TB is summarized in **Table 3**. tNGS demonstrated the highest diagnostic efficacy, with a sensitivity of 76.00%, specificity of 100.00%, PPV of 100.00% and NPV of 87.20%. The AUC was 0.880 (95% CI: 0.777-0.983), and the Kappa value was 0.797, indicating strong agreement with the reference standard. Xpert ranked second, with a sensitivity of 60.00%, specificity of 100.00%, and an AUC of 0.800 (95% CI: 0.674~0.926). DNA detection and culture showed AUC values of 0.780 and 0.740, respectively, both outperforming AFB staining (AUC = 0.648). AFB staining had the lowest sensitivity (32.00%), and a Kappa value of 0.338, suggesting poor consistency with the reference standard.

**Table 3:** Comparison of diagnostic efficacy among different detection methods.

Detection method	Final diagnosis		Sensitivity	Specificity	PPV	NPV	χ²	Kappa	AUC (95%CI)
	Negative	Positive							
tNGS									
Negative	41	6	76.00%	100.00%	100.00%	87.20%	43.757	0.797	0.880(0.777~0.983)
Positive	0	19							
AFB									
Negative	40	17	32.00%	97.60%	88.90%	70.20%	11.524	0.338	0.648(0.503~0.793)
Positive	1	8							
Culture									
Negative	41	13	48.00%	100.00%	100.00%	75.90%	24.053	0.534	0.740(0.604~0.876)
Positive	0	12							
DNA									
Negative	41	11	56.00%	100.00%	100.00%	78.80%	29.142	0.613	0.780(0.650~0.910)
Positive	0	14							
Xpert									
Negative	41	10	60.00%	100.00%	100.00%	80.40%	31.835	0.651	0.800(0.674~0.926)
Positive	0	15							

**Figure 1:**
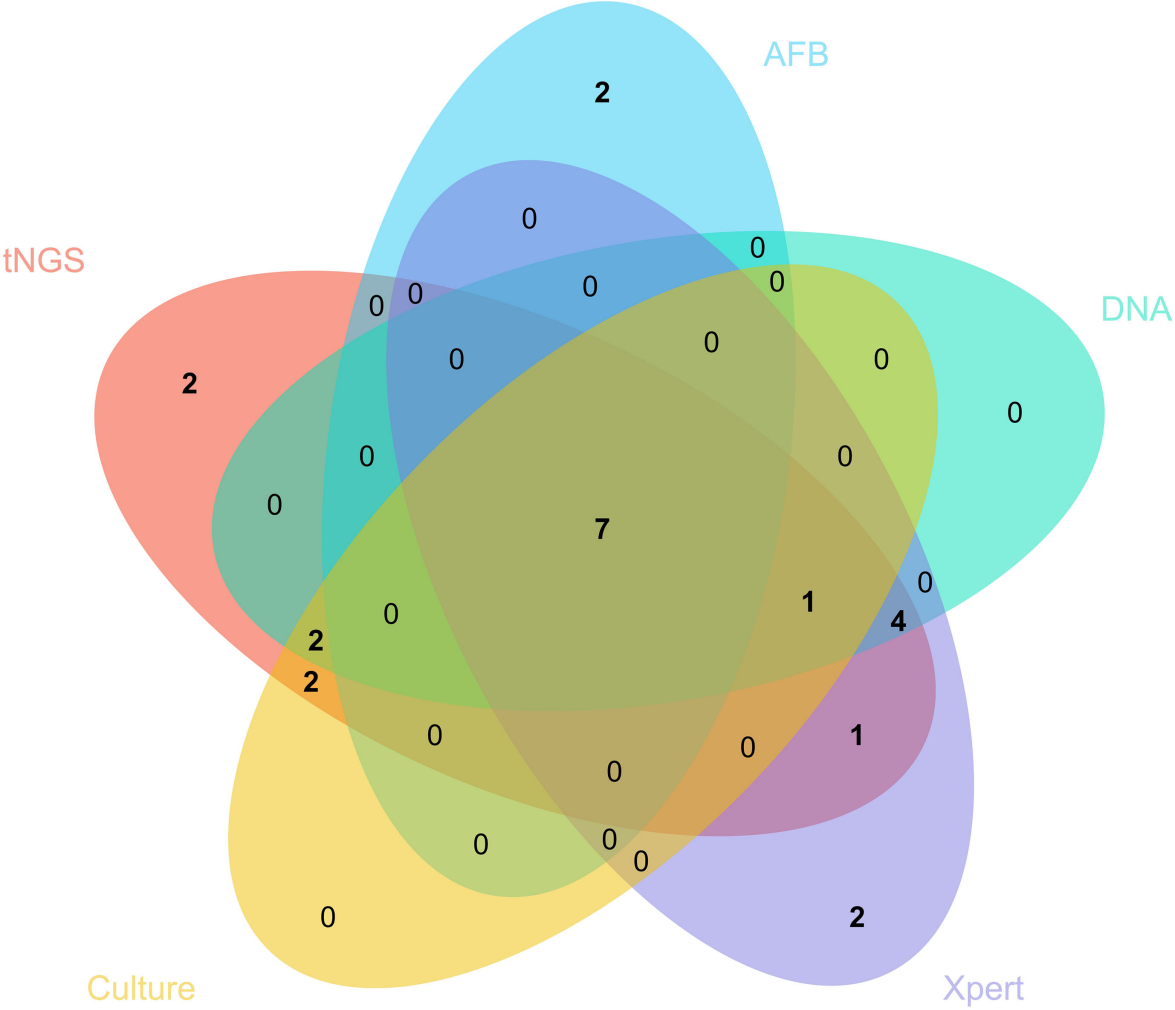
Venn diagram of positive results of five detection methods. Each color represents a detection method.

The Venn diagram in **Figure 1** shows overlapping and unique diagnostic findings among the five methods. Notably, tNGS, AFB and Xpert uniquely identified 2 positive cases. Neither TB-DNA detection nor culture identified unique cases. These findings highlight the complementary advantages of tNGS in enhancing detection sensitivity and case identification compared with traditional and molecular methods.

### 3.3 Combined diagnostic efficacy of tNGS

The combined use of tNGS with other diagnostic methods significantly improved overall diagnostic efficacy (**Table 4**). The combination of tNGS and Xpert achieved the best performance, with the sensitivity of 84.00%, specificity and PPV of 100%, NPV of 91.10%, Kappa value of 0.867 and an AUC of 0.920 (95% CI: 0.833-1.000). These results demonstrate that combining tNGS with Xpert substantially enhances diagnostic accuracy and consistency. Overall, tNGS-based combinations showed strong discriminatory ability and promising clinical value for TB diagnosis in patients with HIV.

**Table 4:** Comparison of diagnostic efficacy of tNGS combined with different detection methods.

Detection method	Final diagnosis		Sensitivity	Specificity	PPV	NPV	χ²	Kappa	AUC (95%CI)
	Negative	Positive							
AFB & tNGS									
Negative	40	5	80.00%	97.60%	95.20%	88.90%	43.065	0.801	0.888(0.789~0.986)
Positive	1	20							
Culture & tNGS									
Negative	41	6	76.00%	100.00%	100.00%	87.20%	43.757	0.797	0.880(0.777~0.983)
Positive	0	19							
DNA & tNGS									
Negative	41	6	76.00%	100.00%	100.00%	87.20%	43.757	0.797	0.880(0.777~0.983)
Positive	0	19							
Xpert & tNGS									
Negative	41	4	84.00%	100.00%	100.00%	91.10%	50.512	0.867	0.920(0.833~1.000)
Positive	0	21							

## 4 Discussion

TB, one of the most lethal infectious diseases globally, presents a core challenge in prevention and control: achieving rapid and accurate etiological diagnosis. Among patients with HIV, early diagnosis is particularly important, as immune suppression accelerates TB progression and often results in atypical clinical manifestations (Wilson et al., 2023; Sossen et al., 2024). However, early diagnosis in this population remains difficult. In recent years, tNGS has emerged as a promising diagnostic strategy, combining the high-throughput capacity of NGS with the efficiency of targeted enrichment (Jin et al., 2025). Although metagenomic next-generation sequencing (mNGS) enables comprehensive analysis of all genetic materials in a sample without culture, its high cost and computational complexity limit routine clinical applications. In contrast, tNGS provides a more practical balance between sensitivity, specificity, and cost-effectiveness. Through pre-designed probes or primers, tNGS enriches specific genomic regions and achieves high-depth sequencing of these loci, thereby reducing turnaround time from sample collection to result reporting (Afonso and Afonso, 2023; Deb et al., 2024). Despite this potential, research on tNGS for TB diagnosis in HIV patients remains limited.

Our findings demonstrate that tNGS offers superior diagnostic performance compared with other methods. In single-detection analysis, tNGS achieved a sensitivity of 70.40%, specificity of 100%, AUC of 0.852, and a Kappa value of 0.748. Notably, the PPV was 100%, indicating that positive results from tNGS are highly reliable, a critical advantage in immunocompromised populations. Although tNGS demonstrated significantly higher sensitivity than traditional methods in this study, eight false-negative results still occurred. The main reasons for this are as follows: First, HIV and MTB co-infection often presents as a paucibacillary disease course, where severely compromised immune function results in extremely low bacterial loads at the lesion site, potentially falling below the detection threshold of tNGS and leading to missed detection (Montales et al., 2015). Previous studies have shown that the bacterial burden of MTB in HIV-infected individuals is significantly lower than that in HIV-uninfected individuals, suggesting a close relationship between immune status and the bacterial load at the lesion site (Hanrahan et al., 2014). Meanwhile, the detection performance of targeted next-generation sequencing is highly concentration-dependent, with a significant decrease in detection success rate in samples with low bacterial loads (Sibandze et al., 2022). Second, the detection performance of tNGS is significantly influenced by sample type. A recent meta-analysis confirmed that BALF exhibits the highest detection sensitivity, whereas some samples in this study (such as blood) have extremely low mycobacterial loads, which may be an important technical reason for the false negatives (Gou et al., 2025). Additionally, as some samples were collected retrospectively, variations in the standardization of collection, storage, and DNA extraction protocols may have affected nucleic acid extraction efficiency and final detection results. These factors collectively explain why, despite its advantages, tNGS may still miss cases in populations with low bacterial loads.

In this study, three patients tested positive by Xpert but negative by tNGS, a discrepancy not uncommon in clinical practice and primarily attributed to the following differences: First, the detection principles differ. Xpert is based on semi-nested real-time fluorescence quantitative PCR, which amplifies a few highly sensitive targets such as IS6110 to prioritize detection sensitivity (Li, 2022). In contrast, tNGS employs targeted sequencing technology to amplify multiple gene regions for broad coverage to meet the demands of drug resistance profiling (Jung, 2026). When bacterial load is low, Xpert may yield a positive result due to efficient amplification of its targets, while tNGS may produce a false negative due to dispersed amplification or insufficient sequencing depth. Second, the operational procedures differ. For tNGS, the process involves multiple independent steps, including sample lysis, nucleic acid extraction, library preparation, targeted amplification, and purification, each of which may result in sample loss. This means that efficiency fluctuations in any step can affect the stability of the final results, leading to a higher risk of cumulative loss, particularly in samples with low bacterial loads. In contrast, Xpert is a fully integrated automated system that combines sample pretreatment, nucleic acid extraction, amplification, and detection within a single closed cartridge, significantly reducing manual operation steps. Its streamlined, fully automated workflow minimizes contamination and reduces the sample risk associated with multi-step procedures (Banada et al., 2010).

In HIV-infected individuals, the bacterial burden of Mycobacterium tuberculosis is typically low, and extrapulmonary TB is more common. Diabetes mellitus can lead to impaired cellular immune function in patients, which may affect the detection rate of MTB, thereby posing a challenge to the sensitivity of diagnostic methods. In contrast, non-tuberculous mycobacteria infections, due to the potential for positive acid-fast staining, may confound the interpretation of smear results for MTB, thereby reducing the specificity of the test. These factors contribute to the limited performance of traditional tests. For example, AFB staining had a sensitivity of only 29.60%, and culture 44.40%, both prone to missed diagnoses. tNGS detected additional cases missed by these methods, including 2 unique positives illustrated in the Venn diagram. While TB-DNA detection and Xpert also achieved 100% specificity, their lower sensitivities suggest reduced utility in immunocompromised patients with low or uneven pathogen loads. The higher sensitivity of tNGS therefore makes it especially valuable in this setting. From a clinical perspective, tNGS demonstrates strong applicability across diverse sample types. In this study, specimens included bronchoalveolar lavage fluid, cerebrospinal fluid, sputum, blood, and tissue, covering both pulmonary and extrapulmonary disease presentations. The stable detection performance of tNGS across these different specimen sources underscores its suitability for complex, heterogeneous cases of HIV/TB co-infection. Previous research has evaluated the diagnostic performance of targeted next-generation sequencing across different specimen types, demonstrating that detection rates were highest in sputum samples, followed by bronchoalveolar lavage fluid and extrapulmonary tissues (which showed comparable performance), then serous effusions, and lowest in lung tissue (Jin et al., 2025). Although that study involved a patient population co-infected with HIV, which may differ from our cohort, the limited overall sample size (n=73) in the present study precludes robust subgroup analyses by sample type due to insufficient statistical power. Consequently, larger-scale studies are warranted to validate these findings and further elucidate the performance of tNGS across diverse specimen types.

Importantly, this study demonstrates that combining tNGS with other diagnostic methods significantly improves overall diagnostic efficacy. When combined with Xpert, sensitivity increased to 81.48%, the AUC rose to 0.907, and the Kappa value increased to 0.846, indicating strong complementarity between the two molecular approaches.

Xpert, recommended by the WHO as an initial diagnostic tool for TB, offers advantages of automation, ease of use, and rapid turnaround (approximately 2 hours), making it ideal for outpatient and emergency settings (Haraka et al., 2021; Dhana et al., 2022; Sossen et al., 2024). However, its limitations stem from dependence on a restricted set of primer-probe sets, which may result in false-negatives in cases with low bacterial loads or rare genetic mutations. tNGS compensates for these shortcomings by employing multiplex PCR to amplify numerous genomic targets, thereby creating a broader “detection net” and reducing the risk of missed diagnoses due to local variations or sampling bias. Our findings suggest that a combined strategy, using Xpert for initial screening followed by tNGS for confirmation, can substantially reduce missed diagnoses and enable more patients to receive timely treatment. The combination of tNGS with culture also achieved 100% specificity and PPV, indicating its value in minimizing false-positives, particularly in cases complicated by NTM co-infection or colonization. This dual-modality approach, rapid molecular testing supplemented by tNGS, shows promise as a future standard for diagnosing complex or atypical TB cases. While culture remains time-consuming, it continues to be indispensable for isolating viable bacteria, performing drug-susceptibility testing, strain identification, and epidemiological tracking. Thus, for patients with positive tNGS results, especially in high drug-resistance settings, culture retains a critical role in guiding individualized chemotherapy and informing public health strategies.

Although tNGS has demonstrated significant advantages in the diagnosis of tuberculosis, its implementation in resource-limited settings still requires careful evaluation of cost-effectiveness and practical feasibility. A programmatic model for the implementation of tNGS in settings with no or low prior sequencing capacity or experience has been proposed, and this model has already been applied in Namibia (de Araujo et al., 2023). Achieving sustainability relies not only on policy support from the WHO and negotiated procurement through the Global Drug Facility, but also on the parallel development of stable laboratory infrastructure, reliable supply chains, and a specialized workforce. In low- and middle-income countries, practical obstacles such as temperature control for reagents, network connectivity, and equipment maintenance are particularly pronounced. However, by drawing on phased implementation experiences from countries like Namibia and gradually enhancing technological accessibility through regional training collaborations, tNGS holds promise for integration into national tuberculosis control programs. This context-specific, phased implementation strategy will maximize its translational value and public health impact in high-burden settings.

Despite confirming the high diagnostic value of tNGS and its synergistic combinations, there are still some limitations. Firstly, the single-center design and relatively small sample size may reduce statistical power and introduce selection bias. Secondly, multiple specimen types were analyzed, the numbers for some (e.g., biopsy tissues and pleural effusions) were too small to draw firm conclusions about robustness across all sample sources. Future research should prioritize large-scale, multi-center, prospective studies to validate the diagnostic value of tNGS across diverse populations, immune statuses, and infection sites. Such efforts will not only provide stronger empirical evidence for integrating tNGS into clinical workflows but also support global public health initiatives aimed at accelerating progress toward the WHO’s End TB Strategy.

## 5 Conclusion

In conclusion, this study confirms that TB diagnosis in patients with HIV, a population with substantial diagnostic challenges, can be significantly improved by tNGS. Compared with conventional methods and other molecular assays, tNGS provided superior diagnostic performance, characterized by high sensitivity, excellent specificity, and adaptability across diverse specimen types. Importantly, combining tNGS with Xpert achieved the optimal balance between sensitivity and specificity, markedly enhancing overall diagnostic accuracy. These findings highlight the clinical utility of integrating tNGS, particularly in combination with Xpert, as a promising strategy to improve early detection and guide timely treatment in HIV/TB co-infected patients.

## Data Availability

The original contributions presented in the study are included in the article/supplementary material. Further inquiries can be directed to the corresponding authors.
